# Qualitative Evaluation of Cancer Survivors’ Experiences of Metacognitive Therapy: A New Perspective on Psychotherapy in Cancer Care

**DOI:** 10.3389/fpsyg.2019.00949

**Published:** 2019-05-01

**Authors:** Mary Gemma Cherry, Peter Salmon, Angela Byrne, Helen Ullmer, Gareth Abbey, Peter L. Fisher

**Affiliations:** ^1^Department of Psychological Sciences, Institute of Psychology, Health and Society, University of Liverpool, Liverpool, United Kingdom; ^2^The Royal Liverpool and Broadgreen University Hospitals NHS Trust, Liverpool, United Kingdom

**Keywords:** metacognition, cancer survivors, anxiety, depression, qualitative

## Abstract

**Background:**

Preliminary evidence suggests that metacognitive therapy (MCT), a brief, process-focused psychological intervention, alleviates distress in cancer survivors. In a longitudinal qualitative study nested in an open trial of MCT for cancer survivors, we explored how patients understood, experienced and applied MCT.

**Methods:**

Patients received six MCT sessions. Consenting patients provided semi-structured interviews post-intervention (*n* = 19), and at 3- and 6-months follow-up (*n* = 14 and 10 respectively). Interviews were audio-recorded and transcribed. Analysis followed a constant comparison approach.

**Results:**

Participants felt “overwhelmed” by worry before starting MCT and doubted that such brief therapy could help. Their accounts focused on feeling “challenged” to think differently by the psychologist. Those completing therapy were enthusiastic about it. They described having learned that thoughts are “only thoughts,” that feelings of worry or sadness are a normal part of life, and that they were in control of whether and how they engaged with thoughts. Consequently, most described a sense of freedom to live free from worry. A minority described being unable to apply MCT to certain thoughts. Two patients who withdrew before completing MCT did not describe having learned what MCT was intended to achieve.

**Conclusion:**

MCT is an acceptable brief intervention for distressed cancer survivors. Feeling challenged to understand the *processes* maintaining their distress was central to their enthusiasm for it, irrespective of their presenting difficulties.

**Implications for Cancer Survivors:**

The complexity of emotional distress in cancer survivors can potentially be addressed using a transdiagnostic model which focuses on the psychological processes which maintain distress.

## Introduction

Advances in early detection and treatment of cancer mean that, despite varying prognoses across different types of cancer, overall around 70% of patients diagnosed with cancer can be treated effectively and live for more than 5 years post-diagnosis (Cancer Research UK, 2017). Therefore there is increasing focus in health care policy and research on the challenges of cancer survivorship (Kline et al., 2018; Rohan et al., 2018). Given the shock of a cancer diagnosis, treatment that can be unpleasant and life-changing, and continued uncertainty over prognosis, it is unsurprising that emotional distress is common in adult cancer survivors, persisting well into survivorship (Deimling et al., 2002; Carreira et al., 2018). Specifically, around a third of cancer survivors experience clinically-significant emotional distress which warrants psychological intervention (Hoffman et al., 2009). Over half also report moderate to high fear of cancer recurrence (Simard et al., 2013).

Psychological distress reduces quality of life and treatment adherence, intensifies physical symptoms and increases healthcare cost (DiMatteo et al., 2000; Carlson and Bultz, 2003, 2004). National and international guidance therefore recommends psychological care for cancer patients who are distressed (National Institute for Health and Care Excellence, 2009, 2017; Butow et al., 2015b; NHS London, 2015). However, meta-analyses evaluating the efficacy of current psychological interventions in cancer populations have yielded only small to modest effect sizes (Rehse and Pukrop, 2003; Demoncada and Feuerstein, 2006; Faller et al., 2013; Temple et al., 2018). One possible explanation is that current approaches to psychological support for cancer patients and survivors tend to focus on the content of their negative thoughts. In particular, the current ‘gold standard’ treatment, cognitive behavioral therapy (CBT; National Institute for Health and Care Excellence, 2009), challenges the validity of intrusive negative thoughts that maintain distress, and of the negative beliefs that underlie those thoughts. However, in a physical health context, such as cancer care, many of the negative thoughts that distress patients reflect accurate fears associated with the disease or its effects, and are therefore less amenable to challenge (Edmondson, 2014). Furthermore, there is evidence that focusing on negative thoughts can itself create a sense of escalating danger that maintains, rather than reduces, emotional distress (Nolen-Hoeksema, 1991; Riskind et al., 2006; Aldao et al., 2010). Therefore psychological approaches which do not primarily dispute the validity of negative thoughts but, instead, help patients to engage less with negative thoughts could be more efficacious in reducing emotional distress in people with physical health conditions such as cancer.

The main “third wave” CBT approaches are Acceptance and Commitment Therapy (ACT; Hayes et al., 2006), Mindfulness-based cognitive therapy (MBCT; Teasdale et al., 1995) and Mindfulness based stress reduction (MBSR; Kabat-Zinn, 2003). However, each of these approaches does involve responding to and dealing with negative thoughts. MBCT has added mindfulness components to CBT, and therefore uses cognitive restructuring, psychoeducation and activity scheduling. The core elements of are MBSR are meditation, yoga and mindful relaxation. ACT has been described as a process based therapy (Hayes et al., 2006) incorporating a range of strategies including forms of meditation, awareness of negative thoughts, and changing avoidant behaviors to more positive ones by reflecting on patients’ valued life aims. Evidence for the efficacy of MBCT and MBSR for emotional distress in cancer patients is not established (Haller et al., 2017). ACT has shown more promise, but its effects are modest (Hulbert-Williams et al., 2018). Although none of these approaches principally involves disputation of negative thoughts, all still require patients to discuss the content of negative thoughts. As mentioned above, key components of CBT are included in MBCT. Both ACT and MBSR require identification of and discussion of the value and benefits of negative thoughts. Each form of engagement with negative thoughts is a type of coping strategy, requiring continued processing of negative thoughts.

Engagement with negative thoughts is, however, the antithesis of the aims of Metacognitive therapy (MCT; Wells, 2000, 2009) which states that any form of coping strategy necessitates conceptual processing. MCT focuses, not on the content of patients’ concerns, but on the metacognitive processes and the beliefs that underlie them. Its premise is that these processes are controllable, and that patients can learn skills to alleviate their distress through a circumscribed, short-term intervention (Wells and Matthews, 1994). MCT is based on the Self-Regulatory Executive Function (S-REF) model (Wells and Matthews, 1994), which is a transdiagnostic model of metacognitive processes and beliefs in psychopathology. This model states that emotional distress becomes persistent when stored metacognitive beliefs guide an individual to respond to commonly-occurring negative thoughts and feelings in a particular way. This is termed the cognitive-attentional syndrome (CAS), and involves three processes: (i) perseverative thinking (i.e., worry and rumination); (ii) threat monitoring (i.e., focusing attention on potentially threatening thoughts, emotions or bodily sensations); and (iii) maladaptive coping strategies (such as avoidance and reassurance seeking). Common concerns in cancer patients are fear of recurrence or functional limitations associated with the disease and treatment. MCT does not challenge the content of these thoughts or use strategies which promote further engagement with those thoughts. Instead patients would be helped to understand the deleterious and counterproductive effects of the CAS, therefore enhancing motivation to suspend worry and rumination. Simultaneously, metacognitive beliefs about the uncontrollability of worry and rumination would be challenged. Modifying negative metacognitive beliefs is central to MCT because, as long as patients believe that perseverative thinking is uncontrollable, they will not try to control it.

Cross-sectional and prospective studies indicate that metacognitive beliefs are associated with emotional distress and fear of cancer recurrence in adult cancer survivors (Thewes et al., 2013; Butow et al., 2015a; Cook et al., 2015a,b) thus supporting the ‘fit’ of the S-REF model to this population. Furthermore, case series and open trials suggest that brief MCT is an effective and acceptable intervention for depression, anxiety and trauma symptoms in cancer survivors (McNicol et al., 2012; Fisher et al., 2015, 2017, 2019) with gains maintained for most patients through to 6-month follow-up (Fisher et al., 2015, 2019). Although these findings point to the need for controlled evaluations of MCT, additional kinds of evidence are needed before progressing to more definitive trials. Qualitative methods can provide information about interventions that trials, alone, cannot. In particular, qualitative research can show how patients understand and experience an intervention (i.e., its ‘face validity’) and can help to understand why an intervention was successful (or not) in individual patients. Qualitative methods can also provide patients’ perspectives on how an intervention was delivered, thus enhancing understanding of its transferability between clinical contexts (O’Cathain et al., 2013). The UK Medical Research Council therefore recommends that researchers use qualitative methods both to understand trial findings and to inform future intervention development, delivery and evaluation (Craig et al., 2008; Elliott, 2010; Moore et al., 2015).

In this study, we report the findings of qualitative research nested in an open trial of MCT for anxiety and depression in adult cancer survivors (Fisher et al., 2019). In this trial, patients received six 1-h sessions of MCT, delivered weekly. Patients completed measures of emotional distress, quality of life, worry/rumination, fear of cancer recurrence and metacognitive beliefs post-intervention, and at 3- and 6-month follow-up (Fisher et al., 2019). We also interviewed consenting patients at each time point, to explore qualitatively: (i) how they understood and experienced the intervention; (ii) once treatment ended, how, and to what extent over the follow-up period, did patients transfer what they had learned across the range of emotional challenges arising during survivorship; and (iii) what characterized any patients who did not benefit? Here we report this longitudinal qualitative study.

## Materials and Methods

Ethical approval was provided by the National Health Service North West Research Ethics Committee (reference 15/NW/0820).

### Participants

Participants were patients referred to an adult clinical psycho-oncology service in the north of England, who (i) had initiated MCT in the context of open trial of MCT for anxiety and depression (Fisher et al., 2019), and (ii) whom we could categorize as clinically improved or not (see below). Inclusion criteria for entering the open trial were that participants: (i) scored ≥ 15 on the Hospital Anxiety and Depression Scale total (HADS; Zigmond and Snaith, 1983); (ii) had been diagnosed with cancer ≥ 6 months previously; (iii) were aged 18 years or over; (iv) had completed acute medical treatment for cancer (i.e., chemotherapy, radiotherapy, surgery); (v) were not receiving concurrent psychological treatment; (vi) were not actively suicidal; (vii) reported no current substance use; (viii) were not experiencing a psychotic or organic illness; (ix) were free from psychotropic medication or on a stable dose for at least 8 weeks; and (x) were able to speak and understand English. Briefly, participation involved attending 6 1-h sessions of MCT delivered over a 10-week period by AB, PF or one other female clinical psychologist (LF). PF supervised AB and LF. After treatment, participants were followed up at 3 and 6 months; no additional treatment was delivered during the follow-up period. Participants completed self-report questionnaires assessing a range of outcomes, including anxiety and depression, worry/rumination, fear of cancer recurrence and metacognitive beliefs, pre-intervention, post-intervention and at 3-and 6-month follow-up. All questionnaires including the primary outcome (HADS-Total) were completed in face-to-face interviews and used pencil and paper format. For full details of each questionnaire see the report of the open trial (Fisher et al., 2019).

In total, 43 consecutive referrals who appeared to meet inclusion criteria for the open trial were given further information about the trial. Of these, 33 agreed to be assessed for eligibility. Twenty-seven participants began the trial, all of whom consented to being approached for qualitative interviews post-treatment and at 3- and 6-months follow-up. Our criterion for clinically significant improvement was ≥ 6 points reduction in the HADS total score post-therapy and at 3- and 6-month follow up by comparison with pre-therapy (Jacobson and Truax, 1991). Twenty participants completed treatment, of whom 19 provided 3- and 6-month follow-up data and could therefore be categorized as improved or not. Of these 19, 15 met our criterion for improvement.

### Procedure

We invited all 19 participants who had completed therapy and whom we could categorize as improved or not to participate in this qualitative study. We also made strenuous efforts to recruit the seven participants who did not complete therapy, whom we regarded as potentially key informants. HU, AB^1^ or GA interviewed consenting participants privately in a University office, hospital consulting room or by telephone, as each patient preferred. Interviews, loosely structured by an interview guide, were conversational, using open questions and prompts to facilitate participants’ talk, with closed questions probing specific points. Pace of the interview and sequencing of specific topics therefore depended on each participant. In general, after reviewing the participant’s cancer journey, the interviewer asked about emotional problems around the time of entry to the trial, expectations of MCT before starting therapy, and their experience, understanding and evaluation of MCT as therapy continued. To avoid generalized or normative accounts, the interviewer prompted participants to focus on specific sessions, and specific experiences in those sessions, and to recount in detail how they addressed specific emotional challenges during the period of therapy and, for follow-up interviews, subsequently. For participants who withdrew prematurely, the interviewer explained that we had much to learn from those who decided that MCT was not appropriate for them at that time, and explored the reasons for the participant’s decision as well as their expectations and experience of MCT. Interviews lasted 7–92 min (mean 40 min).

Interviews were audio-recorded with participants’ permission, transcribed verbatim and anonymised. Participants were allocated a unique code number to preserve their anonymity. Analysis focused first on the participants who met our criterion for clinically significant improvement. Analysis was inductive in describing salient features of participants’ accounts, rather than applying an *a priori* categorization such as offered by the interview guide or metacognitive theory. Specifically, we identified aspects of their accounts which showed strong commonalities or, conversely, marked divergences.

Analysis drew on a pluralist qualitative approach (Salmon and Young, 2018), in which we sought ‘methodological integrity’ by adopting practices that ensured fidelity to the data and utility for the research question (Levitt et al., 2017). Analysis proceeded in parallel with interviews. MC led analysis in frequent discussion with PS and PF, who read all the transcripts over the course of analysis; other authors read selected transcripts or extracts. MC, PS, and PF began by developing a preliminary thematic framework based on the first five participants’ post-treatment interviews. This was tested and refined in discussion and by reference to new transcripts so that the analysis developed iteratively, following a constant comparative approach (Fram, 2013). We used Microsoft Word to label and organize text using inductive headings that evolved over the analysis (Pelle, 2004). Analysis was based initially on the post-treatment interviews, drawing on follow-up interviews as it proceeded and as they became available. The resulting framework provided a starting point for incorporating data from participants who did not meet our criterion for clinically-significant improvement and those who did not complete MCT. During the final stages of analysis, the analysis was tested and further developed by discussion amongst all authors. Throughout analysis, we took a ‘disputational’ approach, whereby we identified and developed competing interpretations and tested their validity by argumentation amongst the group that was grounded in the evidence of the transcripts. As analysis proceeded, we continually judged it according to consensus validity (through debate, it should satisfy all authors; Stiles, 1989), reflexive validity (it should change authors’ initial and subsequent views; Stiles, 1989), catalytic validity (it should have potential practice implications; Stiles, 1989; Kincheloe and McLaren, 2000), and theoretical validity (it should connect with and helpt to test and develop existing theory; Stiles, 1993; Kincheloe and McLaren, 2000). Analysis ended when we judged that the account we produced met these criteria and when further discussion and reference to the data did not appreciably modify it. The team encompassed a range of backgrounds, allowing us to use these different perspectives to inform “investigator triangulation” in analysis (Patton, 2015).

GA and HU are research psychologists with experience in delivering CBT. AB, MC, PF, and PS are clinical psychologists; MC has researched emotional processes in health care; AB has researched patient perspectives in cancer care and is trained in MCT. PF and PS have expertise, respectively, in applying MCT in physical health populations and in studying patients’ perspectives in cancer care. By drawing on these different perspectives we sought to ensure that analysis was inductive and robust, while subsequently interpreting its specific relevance to MCT.

Representative extracts from interviews illustrate the main findings, below. Italicized text indicates direct quotes. For extended quotations, participants are identified by participant number and assessment occasion: 0, 3, 6 indicating immediately and 3- or 6-months post-therapy. Square parentheses indicate explanatory comments; ellipses indicate omitted talk. We first report those participants who met our criterion for clinically-significant improvement; then we report those who did not complete therapy or who completed it but without meeting our criterion for improvement.

## Results

### Sample Characteristics

Of the 19 participants who completed treatment and could be categorized as improved or not, 17 agreed to be interviewed post-treatment. Of these, 13 met the criterion for clinically-significant improvement. At 3- and 6-months follow-up, 14 and 10 participants respectively, could be contacted and agreed to be interviewed. Of the seven participants who did not complete MCT, two, who had attended one and three sessions respectively, provided a brief telephone interview after withdrawing from the trial.

Most participants were female (*n* = 17) and White British (*n* = 18). Ten participants had been diagnosed with breast cancer. Most (*n* = 17) had undergone surgery, chemotherapy and/or radiotherapy. Fifteen participants had previous experience of psychological therapy; of these, eight had attended counseling, and an additional six had had CBT. Time since completing acute medical treatment ranged from 3 to 78 months. Participants’ pre-treatment HADS scores ranged from 20 to 38, with a mean of 25.47 and standard deviation of 6.33. Table 1 displays participants’ demographic and clinical characteristics and HADS scores.

**Table 1:** Patient characteristics.

ID	Sex	Age	Cancer (Stage)	Treatment received	Prior therapy	Time since completing treatment	HADS		Categorization	Interview points
							Pre/Post	3 m/6 m		
1	F	55–59	Breast (0)	Surgery	Couns’	14 m	21/11	07/06	Improved completer	Post; 3 m
2	F	25–29	Breast (I)	CTX, surgery	CBT	3 m	28/16	11/11	Improved completer	Post; 3 m
3	F	30–34	Sarcoma (II; R)	CTX, RTX, surgery	Couns’	8 m	18/03	01/01	Improved completer	Post; 3 m; 6 m
6	M	65–69	Haem (N/A)	Other	CBT	48 m	23/03	00/00	Improved completer	Post
7	F	60–64	Breast (III)	CTX, RTX, surgery	None	7 m	21/03	01/03	Improved completer	Post; 3 m
9	F	60–64	Ocular (II)	RTX	None	14 m	26/05	01/03	Improved completer	Post; 3 m; 6 m
11	F	50–54	Breast (I)	CTX, surgery	Couns’	78 m	38/33	14/11	Non-improved completer	Post
12	F	60–64	Breast (II)	CTX, surgery	Couns’	30 m	30/10	13/08	Improved completer	Post; 3 m; 6 m
13^a^	F	45–49	Ovarian (I)	Surgery	Couns’	34 m	38/–	–	Non-completer	After withdrawal
14^b^	M	50–54	Haem (N/A)	CTX	Couns’	18 m	22/–	–	Non-completer	After withdrawal
15	F	60–64	Breast (unknown)	RTX, surgery	Couns’	36 m	24/08	09/12	Improved completer	Post; 3 m; 6 m
16	F	30–34	Breast (II)	CTX, RTX, Surgery	CBT	34 m	22/06	31/20	Non-improved completer	Post
18	F	50–54	Colorectal (I)	Surgery	CBT	13 m	32/20	22/25	Improved completer	Post; 3 m; 6 m
19	F	50–54	Haem (N/A)	TKI	MCT	24 m	19/07	08/06	Improved completer	Post; 3 m; 6 m
21	F	50–54	Breast (II)	CTX, RTX, surgery	CBT, Gestalt	24 m	18/10	09/18	Non-improved completer	Post; 3 m; 6 m
23	F	45–49	Ovarian (III)	CTX, surgery	None	14 m	24/08	16/05	Improved completer	Post; 3 m; 6 m
27	F	35–39	Breast (II)	CTX, RTX, surgery	None	16 m	23/11	15/14	Improved completer	Post; 3 m; 6 m
30	F	60–64	Breast (III; R)	CTX, RTX, surgery	CBT	13 m	35/22	27/33	Non-improved completer	Post; 3 m
31	F	55–59	Ovarian (II)	CTX, surgery	Couns’	24 m	22/00	02/01	Improved completer	Post; 3 m; 6 m

*^a^Completed one session; ^b^completed three sessions; CBT, cognitive behavioral therapy; Couns’, counseling; CTX, chemotherapy; F, female; Gynae, gynecological; Haem, hematological; M, male; m, month; Pre, pre-therapy; Post, post-therapy; R, recurrence; RTX, radiotherapy; TKI, tyrosine-kinase inhibitor.*

#### Participants Who Completed Therapy and Consistently Improved

In summary, these participants described their starting point of intense worry, the challenge of MCT, learning a new relationship with their thoughts, and thereby gaining a feeling of freedom to live their lives.

##### The starting point: ‘caught in a spiral of worry’

When recalling life before starting MCT, participants consistently described being “*caught in a spiral of worry,”* going “*round and round in circles,”* “*suffocating”* or becoming “*utterly exhausted,” “helpless,” “hopeless”* and “*engulfed”* or “*overwhelmed”* by worry. Worry centered on cancer and its consequences for themselves and others, and on what could be done to mitigate risk of recurrence. For many, worrying was linked to extensive Internet searching. For example, P23/0 described “*looking at the death statistics to try and work out where I thought I was on the graph”* even though *“it was just doing me no good. Absolutely nothing from doing that was doing me any use at all.”* Every participant worried also about concerns distinct from cancer, including financial problems, family members’ illness and neighbor disputes. P12/3 illustrated how participants described worrying to try to mitigate challenges: “*The way I got through my day was having everything controlled…What I used to do was overthink what was coming. ‘This might happen, this might happen, Oh this could happen.’ So I would try to work out what may or may not happen so that if it did happen then I’d pre-thought it through and…I’d be able to handle it better.*” Some participants, like P3, described worrying about worry: “*I’ve said it before to [oncologist] and to [nurse]: ‘I’m convinced I’m making myself ill. I’m making myself ill because I am stressing that much and that isn’t good for the cancer.”*

##### Doubt and challenge in learning MCT

###### ‘My worry is part of me, and MCT will be too brief to change me’

A few participants, like P15/0, recalled being “*curious*” and “*hopeful*” before starting MCT, or thought that “*it’s got to be better than nothing”* (P18/0). In general, however, participants began MCT *“skeptical as to such a short sort of program being able to fix me…I felt like my anxiety was just intrinsically part of who I was”* (P2/0).

Participants linked their skepticism to the challenge that MCT presented to their belief that they could not control worrying. P12/0 thought the explanation of MCT at the start of therapy “*was almost quite naïve…too simplistic to assume that you can switch it [worry] off.”* P31/0 described MCT as “*scary initially, because I was always under the impression that I couldn’t control these, these thoughts and I had to pay attention to them.*” Several participants had received therapy previously, and spontaneously cited their experience to support their belief that they could not change (Table 2).

**Table 2:** Patients’ accounts of how MCT compared with therapy that they had received previously.

‘I’ll never be able to change’	Except for one patient who recalled thinking that MCT *“could be quite good because I’d just been introduced into mindfulness”* (P19/0), patients’ experiences of previous therapy had reinforced beliefs that they could not change and that MCT would therefore not help. For instance, P2/0 explained that CBT *“hadn’t changed me, so [when approached about MCT], I think I was quite skeptical as to such a short sort of program being able to fix me.*” P16 had attended “*over 20*” sessions of CBT. *“When [the psychologist] said it [MCT] was six sessions I was like, ‘What’s that going to do?’ I was really skeptical at the start, but now it’s just, it’s just amazing”* (P16/0). Patients described losing their skepticism when they encountered what MCT involved. For instance, P18 had at first been “*pessimistic”* because, with CBT, *“I thought ‘I’m getting worse. I’m feeling worse, here. It’s making me more depressed.”* By contrast, over the initial sessions of MCT, the psychologist’s questioning about habits of rumination – “*Did I dwell on it? Did it make me feel worse?” –* and her “*good explanation*” that *“because you’re ruminating a lot and you’re going deeper and deeper into your thoughts, it’s actually causing more depression”* and that *“they’re just thoughts, and you can just take a hold of those thoughts and move them to one side”* gave P18 *“an insight of what was achievable and what was to come with the following sessions”* (P18/0).
‘MCT is challenging, not just a temporary support’	Patients felt challenged by the psychologist, contrasting this experience with previous supportive relationships with therapists, psychologists or counselors. Patients felt that feeling challenged led to more open and honest discussions, as P3/0 illustrated: “*[In counseling] it would be, ‘How’s your week been? How are you feeling?’, that’s it, whereas this [MCT] was more specific, like, ‘Any thoughts, anything you have really thought about that has led you to excessive worrying?’ … I just felt like [psychologist] wanted to understand the problem not just in general how I was feeling. That’s what made it really stand out… I’d find I would tell [psychologist] things… a few things that I have never said out loud before… [Psychologist] would really quiz me on things as well and wouldn’t let me drop it. She’d say, ‘But why, but why, can we come back to that?’ and then I’d think, ‘Well, I can’t avoid it anymore’ so then I’d be honest about it”* (P3/0). A second consequence, that P15 illustrated, was that the benefits of seeing the psychologist persisted. Post-treatment, she described MCT as *“something you can take away. I mean there’s other good therapies but it’s only effective while you’re there, or while you’ve got that support there. Whereas this is, in a way, meant to be empowering.”* At 6-month follow-up, she explained: *“I have had PCC [person centered counseling], which at the time was good because it was somewhere to get an awful lot off my chest… [but] I think this [MCT] is more useful because you can take it away, and you are in charge of it… This [MCT] gives you a strategy for calming yourself down, keeping yourself on an even keel, and feeling more in control of your own life really, instead of other people, controlling things… I think the PCC was helpful, but it was a temporary relief”* (P15/6).
‘A new relationship with thoughts’	After MCT, patients described having learned to accept troubling thoughts and feelings as normal rather than, as previous therapy or counseling seemed to require, as experiences to be disavowed. As such, patients saw MCT’s emphasis on *not* engaging with negative thoughts and feelings as better addressing the root cause of their distress. For instance, P12/0 had experienced two cancers and also family deaths from cancer, and feared for her children were she to die: “*Ever since the second diagnosis… I’ve been trying to use my brain to control this body…You’re thinking ‘Oh no don’t get upset’ whereas actually, you know, it’s normal to be upset… I think the therapy tries to show you that there are stuff and feelings that are part of being a human being and that that’s normal to do... I think it’s recognizing the feelings that you get that are genuine and understandable.”* By contrast, she recalled a counselor asking her to *“imagine it [upsetting thought] floating down the stream and stuff and I, I couldn’t do all that… I just thought that’s ridiculous… I didn’t want my feelings belittled. I didn’t want them not taken seriously.”* Similarly, P16/0 explained that “*what she [CBT therapist] was doing was the equivalent of just putting a bit of gauze on an open wound kind of thing, do, rather than actually try and heal…”,* whereas MCT meant “*stopping everything before it went from thought to either worry or rumination… It was actually cutting it off right at source rather than… with the CBT you’ve got to go into it and that was what gave me a lot of hope for this [MCT] working, and I thought it made a lot of sense. You’ve always got to go back and, and analyze them [thoughts] and think about them when doing the CBT, and that was half the, or even more than half of the problem”* (P16/0).

###### The psychologist provides ‘challenge’, not ‘support’

Once participants started MCT, they consistently experienced it as *“challenging,” “mentally demanding,” “hard work,” “tiring”* or even *“grueling.”* For example P2/0 recalled her first session as “*really exhausting… [I left] feeling like I’d like ran a marathon*…*I remember crying a lot and it was very challenging. I think I was really exhausted at the end of it…because it just completely challenged a lot of beliefs.”* Similarly, P7/3 described MCT as “*like retraining your brain rather than learning something…And in a way going to the gym is hard work and you don’t want to do it. It’s the same I think*.”

Given their doubts, and the challenge associated with MCT, participants consistently attributed their continued participation to the psychologist. When explaining how the psychologist helped, only P1/0 referred to feeling emotionally supported, recalling the psychologist explaining that P1 was “*not the only one”* to have feelings of guilt and anger, making her feel *“comfortable”* and “*relaxed”* and becoming a *“crutch.”* By contrast, all other participants, and also P1/0 elsewhere in her interview, emphasized the psychologist’s didactic or “*challenging”* role, describing her “*telling,”* “*teaching*” or “*explaining.*” Several referred explicitly to challenging questioning; for instance when *“I said that…I couldn’t control the worry. And [psychologist] was asking me things like ‘Do you worry when you sleep? Do you worry when you’re doing other things? Do you worry when you’re with your daughter?’…I’m like, ‘Well, no, no I don’t do it then.’ And she said, ‘But you say here that you worry 100%, all the time, non-stop.’... And then she would say things, ‘Well who’s controlling the worry?’ ‘Who is the one who is worrying and who is controlling it?’ And I thought, ‘It’s me’.”* (P9/0). Several participants contrasted their experience of MCT with prior experience of counseling (Table 2), emphasizing in particular the psychologist’s “*structured*” and “*challenging*” approach and explaining that benefits of MCT therefore endured beyond the relationship with the psychologist. None described wanting more support or understanding from the psychologist, and none recounted any feelings resembling abandonment at the end of therapy.

##### A new relationship with thoughts: ‘they’re only thoughts’

Participants who met the criterion for improvement all recounted learning a new relationship with their thoughts, with three defining features: they recognized that thoughts would pass if they allowed them to, and were not problems in themselves to be addressed; they explained that feelings of worry or sadness were a normal part of life that they could accept rather than fight; and they felt in control of whether and how they engaged with their thoughts.

###### ‘Let thoughts pass’

Participants consistently described having learned, when thoughts that used to trigger worry and rumination arose, “*don’t fight it*” or “*don’t latch onto it*” and, instead, “*let it pass*”; “*it’s only a thought.*” As P6/0 explained, “*the biggest lesson I’ve learned is when your thoughts come to you, you don’t try to dispel it. You don’t fight…don’t try and solve it. You just…let it pass.”* Participants attributed their ability not to engage with these thoughts or images to appreciating that they are *“like other thoughts in your head all through the day… [that] will go, will pass*” (P7/0). They recognized that the thoughts were not in themselves problems that required solutions, nor were they dangerous. For P12/0, *“my biggest battle was accepting that… the thoughts [about cancer recurrence] won’t cause cancer.”*

Participants recounted insights about worrying, also. They consistently described learning that worry was not useful, for instance, that *“worrying about something is not going to prevent it happening…I believe that now”* (P1/0). Some described having learned not to be frightened of their own worry. For instance, P2/3 described having learned “*that I didn’t have to be scared of the feelings when they came…It was kind of like a light switch that was sort of like, I don’t have to be scared of that [worry] anymore and, you know, it’s not going to hurt me.”*

###### ‘It’s normal to have upsetting thoughts at times’

Participants learned to recognize that many of the thoughts and feelings that previously triggered worry and rumination concerned real challenges or memories that it was natural to feel upset or unhappy about, but that they need not worry about feeling upset or unhappy. For instance, P12/3 explained that, “*[Before therapy], I always used to try and say to myself ‘Oh snap out of it [low mood], stop doing it [worrying],’ you know, and was very negative towards myself, almost feeling was though I was a failure because I couldn’t…control the thoughts. And I think now I’m much more accepting that it’s normal to have these thoughts that will pop into your head.”* Most participants experienced distressing thoughts associated with their continuing vulnerability to cancer throughout the period of therapy and follow-up, particularly when anticipating scans or scan results and, after MCT, they consistently recognized that it was normal to be upset in those circumstances and that they need not worry about being upset. For instance, P3/0 reflected that “*I think I’m going to be upset about [a forthcoming scan]…because I think it’s natural to feel that if you walk into a room and know you could be getting a life-changing decision. I feel OK about it to be honest with you.*”

However, participants recounted diverse challenges arising during the period of therapy and through follow-up that were not directly related to their cancer, including relationship problems, and serious illness in family or friends. Those who met our criterion for improvement consistently described having learned to accept upsetting thoughts at such times as normal responses to such challenges that did not need to be resisted or controlled. For instance, in recounting diverse challenges at 3-months follow-up, including a friend’s death, becoming pregnant and moving house, P2/3 explained that *“As the things have happened I’ve just let them…My friend dying was the big big big big one, and you know that’ll stay with me but…I’ve just kind of existed with it…I haven’t really fought against anything... It’s not that I haven’t had them [low moments], I just haven’t done my habitual, sort of, catastrophizing.”* Similarly, P3/0 spoke about her sadness at learning that a relative was incurably ill: “*I appreciated the fact I could have a normal emotion about it and I didn’t have to control it…I didn’t try and stop the sadness because I thought ‘Well you know at the end of the day it is a sad situation and I’m allowed to be sad’.”*

Participants who had received previous psychological therapy spontaneously and strikingly contrasted their new ability to accept troubling thoughts and feelings as normal with the disavowal of those thoughts and feelings that they associated with previous therapy (Table 2).

###### ‘I can control how I react to my thoughts’

Whereas participants all described having felt “*out of control”* of worry before MCT, they consistently referred to having gained a sense of agency after completing therapy. They used phrases like *“I have decided,” “I’m in control”* or *“I’m not going to do that”* to describe their “*decision”* not to engage with thoughts or feelings that used to trigger worry. P9/3 explained that *“I wouldn’t say I’m in control of my thoughts, because I’ve got no control over them, but I can control over what I do about them.”* P15/0 described how MCT *“gave me the awareness that I could choose...to react differently [to thoughts],”* explaining that “*one [exercise] that was very helpful for me was the ‘You don’t have to pick up the phone when it rings.’ I found that quite useful, or ‘Actually I can deal with that [thought] later, I don’t have to deal with it right now, so I can put it on the backburner’... It’s the realization you don’t have to go with it...there’s a choice involved in how you do it.”* Participants spoke about the confidence that this sense of control gave them in an inherently uncertain context in which upsetting or challenging events arose unpredictably. For instance, at 3-month follow-up, P1’s sense of vulnerability to recurrence of her breast cancer had been heightened by two friends’ recent discoveries of incurable metastases associated with their own breast cancer. She felt that the MCT “*is helping me to control my mind…All this mental therapy is preparing me for the medical side of it for when it [her own cancer] comes back…I feel like I can trust…the mental side of it but I don’t feel I can trust the medical side of it.*” Thoughts about her vulnerability were therefore “*frustrating*” rather than, as previously, “*overwhelming.*”

Participants sense of their own agency extended retrospectively; that is, after therapy, they appreciated that their previous distress had been “*self-generated*”: “*You’ve done this to yourself. It’s not an illness.*” However, no participant described feeling blame or guilt. They were protected because “*I wasn’t realizing that that’s what it was”* (P2/0), but also because MCT explicitly taught them not to engage with such thoughts. Several participants explicitly contrasted this understanding from MCT with the culpability that they felt previous therapy had implied (Table 2).

##### The benefit of therapy: ‘I can live my life now’

After MCT, participants who improved consistently reported feeling “*happier*” and “*free”* to “*enjoy life”* and “*live instead of function.*” Central to this newfound freedom was deciding what they could and could not influence. Participants described not trying to pre-empt events they could not change; that is, “*taking things as they come.”* However, they had not simply learned to acquiesce to events. Instead, they describing feeling “*free to make decisions”* or “*putting myself first*” where they could.

###### ‘I’ll take things as they come’

Participants who met our criterion for clinically-significant improvement consistently described accepting that they had to live with uncertainty but need not worry about possibilities that they previously would have worried about. For instance, P31/6 reflected that “*I don’t really dwell on what could happen…If it happens, it happens…You can’t say ‘Look I’ve had enough of this, don’t give me no more cancer’ or any of that…You just take it as it comes.”* Most strikingly, participants took this attitude to the possibility that scans or investigations would reveal cancer recurrence. For instance, at 3-month follow up, P3/3 referred to her forthcoming scan: “*I can’t change the scan [outcome]…If there’s anything there then I’ll deal with it, I’ll find out what the treatment plan is and that’s it. I won’t worry about it…I’m not living if I’m worrying or stressing about it.*” Similarly, at 6 months follow-up, P9/6, whose cytogenetic test results put her at high risk of recurrence, described waiting for recent scan results: “*[Thought of recurrence] is just a thought. There’s no point in me worrying about this. This is a thought…And for the whole week that was popping in: ‘Someone’s looking at this MRI. The radiologist will be looking at this now.’ That’s a thought, that’s OK…It’s just a thought. And whatever the outcome is of this MRI, I will deal with it then.”* Several participants described developing reasoned strategies for when to seek investigation for symptoms that might signify recurrence, without the intense worry that would previously have surrounded reminders of vulnerability. As P3/6 explained: *“If I think I have a twinge I just do my normal checks and just think ‘that’s nothing to worry about’…I had a twinge the other day. And I did say to my husband ‘Listen I’ve had a twinge, and if it’s still there in four days,’ you know, give myself a deadline, ‘I’m going to get onto them’. And I’ve not had it since, so I was just quite sensible about it…The fact that it’s been there one day, I’m not going to worry about it.”*

###### ‘I’m free to put myself first’

After MCT, participants described “*putting myself first*” where they could, and having time to do ‘*the practical things… [which] previously I didn’t really have the time to do because most of the time was spent worrying, feeling I had to worry” (P9/3).* Several recounted major life changes. For instance, at 3 months, P23/3 had become able to “*fill my thoughts with better things like the future”*; at 6 months she had “*made that decision [to resign a highly paid but demanding job]…because I’m actually now thinking about the future which I wasn’t [before MCT].*” In the same vein, participants reported booking holidays that they had previously avoided lest forthcoming scans revealed problems (P12/3; P3/6), taking on new, more challenging, roles at work (P3/6), allocating more time to friends and family (P9/6; P19/6; P31/6), and deciding to prioritize their own needs rather than acquiescing to others’ demands (P12/6).

#### Participants Who Did Not Complete Therapy or Did Not Consistently Improve

Those participants who did not complete therapy, or who completed it but without meeting our criterion for clinically-significant improvement, formed a heterogeneous set, both in their clinical response to MCT and in their interviews. All described the same starting point as those participants who made clinically-significant improvement; that is, against a background of feeling that life was dominated and constrained by worry, they recalled being initially skeptical about MCT and described early sessions as “*hard work”* because the psychologist challenged entrenched beliefs about worry. However, neither of the participants who withdrew from treatment (P13, P14) indicated any understanding of MCT, describing it simply as “*to help me understand the cancer side of things and to show I wasn’t alone”* (P13/0) or “*trying to boost my self-esteem”* (P14/0). They each described MCT as too ‘*stressful’* or ‘*intense.’* By contrast, all four participants who completed MCT, but without meeting our criterion for improvement, appeared to understand the model, for instance summarizing its goal as “*not to have dialogue with thoughts anymore”* (P30/0) because *“the whole process of engaging, either through rumination or worry, is then you do end up with further worry, further rumination, more thoughts, a whole process of thoughts that lead you down a path to depression, basically”* (P21/0). Similarly, their accounts of what they had learned about MCT resembled those of participants that were successfully treated: “*I can’t control my thoughts but I can control what I do with them and, and whether worry or rumination happens with them”* (P16/0). However, these participants found it difficult to apply what they had learned. Two gave clues as to what was difficult, describing successfully disengaging from some thoughts but not others, particularly ones related to family roles. For instance, P11 described having “*more powerful thoughts [concerning her children’s long-term health conditions]…that I can’t seem to just let go of…that I’m finding difficult to be able to just go, ‘Yeah, it’s a thought”’* (P11/0). Similarly, although P21 could apply MCT to thoughts about the potential for cancer recurrence, she could not apply it to her angry thoughts about her partner, describing feeling “*adrift*” and “*conflicted*” about the future of her relationship.

## Discussion

### Overview

All participants described feeling consumed by worry before starting therapy, doubting that such brief therapy could help them, and feeling intensely challenged to think differently by the psychologist providing MCT. All those who completed therapy described learning a new relationship with their own thoughts and gaining a sense of freedom to live lives free from worry about cancer and other challenges of survivorship. However, those who did not consistently improve described being unable to apply MCT to particularly troubling thoughts. Two participants who withdrew before completing MCT did not describe having learned what MCT was intended to achieve.

### Results in Relation to Previous Literature

Consistent with the qualitative accounts of most of the participants who completed therapy, findings of the open trial examining the potential of MCT to alleviate emotional distress in adult cancer survivors were promising (Fisher et al., 2019). MCT can be delivered as a transdiagnostic intervention which appears to reduce multiple forms of distress across a heterogenous group of cancer survivors. By contrast with other psychological approaches in cancer care, MCT is the only one which directly focuses on metacognitive beliefs and processes to alleviate distress. Although “third wave” approaches (ACT, MBCT and MBSR) do not predominantly focus on the content of patients’ thoughts, all incorporate greater discussion of cancer related thoughts than does MCT. Furthermore, MCT does not require patients to apply coping strategies which require continued conceptual processing of negative thoughts.

Participants’ awareness of how worry and rumination contributed to emotional distress was the key to their experiences of MCT. Before starting therapy, participants regarded worry as ‘*part of who I was*’ and doubted that it would help them. For some, doubts related to the brief duration of the intervention; for others, prior experience of unsuccessful therapy left them pessimistic. Nevertheless, reservations diminished among participants who continued therapy as they described learning that they need not engage with distressing thoughts. They learned to question their beliefs that worry and rumination were necessary or dangerous and they learned that distressing thoughts were ‘normal’ and need not trigger attempts to control them; that is, participants described becoming effectively ‘socialized’ to the MCT model (Wells, 2009). After receiving MCT, participants were positive beyond polite acquiescence with the interviewer, usually effusively so as they described feeling able to live their lives and make decisions for the future. Contrary to previous qualitative evaluations of cancer patients’ experiences of CBT and relaxation therapy (Hoeck et al., 2017), participants had a clear understanding of the aims of therapy, and no participant indicated an unmet need to address the content of their worries. Indeed, those with previous experience of content-focused therapies contrasted them negatively with MCT. In particular, most participants who completed therapy valued learning to accept negative thoughts and feelings as appropriate responses to negative life events, by contrast with previous CBT or counseling in which they had felt expected to deny the validity of those experiences.

Experiences of the clinical relationship were also in striking contrast with the way in which this is portrayed in psychological care generally, and cancer care particularly, in which the psychologist’s empathy and supportive role are central (Rogers, 1951; Boulton et al., 2001; MacCormack et al., 2001). Participants who completed therapy did not describe relying on their psychologist for support but, rather, valued being *challenged* to think differently and question long-held beliefs about worry and rumination. Moreover, despite the focus of MCT on developing metacognitive awareness rather than a therapeutic alliance, none of the participants who continued therapy indicated any unmet need for the psychologist’s understanding or acceptance, nor did they report feeling ‘abandoned’ at the end of therapy, despite therapy being relatively brief. There is little information regarding how cancer patients feel after other types of psychological therapy, but what there is indicates that feelings of abandonment are a feature of patients’ experiences of CBT and relaxation therapy (MacCormack et al., 2001). Our findings indicate that this is an area that needs further research, particularly given the emphasis placed on the importance of longer-term, supportive therapies in cancer care (Rogers, 1951; Boulton et al., 2001; MacCormack et al., 2001).

Participants who met our criterion for improvement consistently described being able to apply what they had learned beyond the duration of therapy. Those who had received other therapies explicitly contrasted MCT with those experiences of therapy which, they felt, had provided no long-term benefits. In addition, participants described generalizing their ability not to engage with thoughts to those arising across the diverse range of challenges associated with survivorship, from anticipation of impending scans, through fears of recurrence and death, to challenges unconnected with cancer, including relationship problems and other peoples’ illnesses. It seems that, by being challenged to recognize their *absence* of control over some events, but their control over how they responded to thoughts about those events, participants who improved had acquired an understanding that they could apply very generally, without continuing reference to the psychologist. These participants therefore described feeling a sense of empowerment after completing therapy, and consistently referred to gaining a sense of agency and responsibility for their own distress. This extended retrospectively, as participants who improved freely described appreciating that they had been responsible for their distress. However, no participant described feeling blame or guilt over this responsibility; they explained instead that MCT had taught them not to engage with such thoughts. One participant contrasted having learned from MCT to take responsibility for his distress with his experience of CBT, in which he felt both responsible and blamed. However, the only study that, to our knowledge, has examined cancer patients’ experiences of CBT (MacCormack et al., 2001) did not find that feelings of either responsibility or blame were salient features of patients’ experiences. Our findings suggest that the role of agency and control in understanding the effects of psychological therapy in cancer care is an area that needs more research.

Four participants who completed therapy did not meet our criterion for clinically-significant improvement. Although these were indistinguishable from the participants who did meet the criterion in the way they described learning a new relationship with their thoughts, each described failing to generalize what they had learned across the range of thoughts that distressed them. From an MCT perspective, it seems that these participants continued to label certain thoughts as ones they could not, or did not want to, disengage from. Whilst it is possible that psychologists more experienced in delivering MCT could have helped these participants to generalize MCT more widely, there may be certain patient characteristics which militate against generalization but which our study did not identify. Future quantitative studies could investigate this further, for instance by examining whether previous history of psychological morbidity or adversity moderates treatment effects.

Seven patients in the open trial did not complete the course of therapy. Each of the two whom we could interview described MCT as too ‘stressful’ or ‘intense’ for them to continue; both had previously received counseling and spoke about MCT in a way that suggested that they expected a similarly supportive, non-directive intervention. However, we know nothing about the perspectives of the remaining five participants that did not complete therapy; in particular, we do not know whether participants who had no prior experience of counseling also came with these expectations. The possible importance of patients’ expectations of therapy in their readiness to accept MCT needs further research.

### Strengths and Limitations

As a qualitative study of a sample of patients from one UK center, our findings cannot necessarily be generalized. Moreover, our participants were self-selected and mostly White British females, reflecting the profile of referrals to the psycho-oncology service. However, we aimed to minimize researcher selection by offering entry to the open trial to all suitable consecutive patients, and then inviting for interview all participants who completed therapy and could be categorized as improved as well as all those who could be categorized as not improved. Although we made strenuous efforts to interview those who did not complete therapy, we were only able to obtain brief interviews with two from this important group and therefore have little information about why cancer survivors might withdraw from MCT. We thereby recruited a sample of cancer survivors who presented with a diverse range of pre-treatment difficulties including fear of cancer recurrence, post-traumatic stress symptoms, bereavement, depression and anxiety. We did not have ethical approval to interview those that did not consent to the trial. Although our analysis was inevitably shaped by the perceptions and experience of the research team, we strenuously disputed different interpretations of the data and, through our diverse backgrounds in MCT, CBT, and emotional and patient perspectives, brought different viewpoints to the analysis.

### Implications

Our findings can inform randomized evaluations of MCT for distressed cancer survivors. In particular, although MCT was an acceptable intervention for most participants, a quarter did not complete therapy and we have little information about their reasons. To reduce attrition some patients, particularly those with prior experience of supportive counseling, might need to be helped to prepare for the ‘challenging’ nature of MCT. In addition, more attention will be needed to style of delivery, particularly in early sessions. For instance, it may be better to use gentler questioning to challenge beliefs until patients are socialized to the MCT model. Around a fifth of patients who completed treatment were not consistently improved across the post-therapy and followup assessments, and future trials will need to characterize this group further.

Nevertheless, our findings point to the need to question important assumptions that underlie the broader field of psychotherapy in cancer care. First, it is widely assumed that the psychological complexity that cancer survivors present necessitates an integrative framework in which different therapeutic models are combined to match the range of patients’ needs (Boswell et al., 2010; Wells and Fisher, 2015; Curran et al., 2017). For instance, most recently, Curran’s integrated model of anxiety (Curran et al., 2017) proposes that psychologists draw from models including CBT, MCT, ACT, cognitive-processing therapy and existential therapy, when working with cancer survivors experiencing anxiety and fear of cancer recurrence. Our data, instead, point to the potentially broad clinical utility of a single model which helps patients to understand, in an accessible and simple way, *processes* that maintain their emotional distress, irrespective of the nature of their presenting difficulties (Wells, 2009).

Second, our findings challenge the commonly-held belief across psychotherapeutic professions that a therapeutic relationship must be anchored in non-specific, relational Rogerian principles of empathy, supportive listening and trust, and should be prioritized and fostered before meaningful therapeutic change can occur (Rogers, 1951; Boulton et al., 2001; MacCormack et al., 2001). Our participants’ experiences indicate that a therapeutic relationship formed as the patient became socialized to the therapeutic model, and developed as participants witnessed their own progress. Therefore, for most patients, psychologists might not need to address the therapeutic relationship explicitly beyond working in a collaborative and Socratic manner (Byrne et al., 2018). However, these findings arose in the context of MCT, and it remains to be seen whether patients engaging with other process-focused models would have similar experiences.

## Conclusion

Despite the widely accepted importance of using qualitative findings to understand how patients experience interventions (i.e., to understand their ‘face validity’), and to inform future intervention development, evaluation and delivery (Craig et al., 2008), our study is one of few published evaluations of patients’ experiences of psychotherapy in cancer care. Although the findings of this longitudinal qualitative study will inform future controlled trials of MCT, they also more broadly challenge current assumptions about psychotherapy in cancer care. Future qualitative studies are needed to evaluate whether these findings are unique to MCT, or apply more broadly to processed-focused psychotherapeutic approaches.

## Ethics Statement

This study was carried out in accordance with the recommendations of the National Health Service research ethics service with written informed consent from all subjects. All subjects gave written informed consent in accordance with the Declaration of Helsinki. The protocol was approved by the North West Greater Manchester East Research Ethics Committee.

## Author Contributions

PF conceptualized and designed the study. AB, GA, and HU carried out the interviews. AB and HU transcribed interviews and contributed to analysis. MC led the analysis, aided in data analysis and interpretation by PS and PF. MC and PF drafted and revised the manuscript. PS provided detailed feedback on iterations of the manuscript. All authors take responsibility for the integrity of the data and the data analysis.

## Conflict of Interest Statement

The authors declare that the research was conducted in the absence of any commercial or financial relationships that could be construed as a potential conflict of interest.

## Footnotes

1 AB did not interview patients whom she had seen for therapy.
